# Simultaneous acquisition of EEG and NIRS during cognitive tasks for an open access dataset

**DOI:** 10.1038/sdata.2018.3

**Published:** 2018-02-13

**Authors:** Jaeyoung Shin, Alexander von Lühmann, Do-Won Kim, Jan Mehnert, Han-Jeong Hwang, Klaus-Robert Müller

**Affiliations:** 1 Department of Biomedical Engineering, Hanyang University, Seoul 04763, Korea; 2 Machine Learning Group, Berlin Institute of Technology, Berlin 10587, Germany; 3 Department of Biomedical Engineering, Chonnam National University, Yeosu 61186, Korea; 4 Institute of Systems Neuroscience, Medical Center Hamburg-Eppendorf, Hamburg 20246, Germany; 5 Department of Medical IT Convergence Engineering, Kumoh National Institute of Technology, Gumi 39177, Korea; 6 Department of Brain and Cognitive Engineering, Korea University, Seoul 02841, Korea; 7 Max Planck Institute for Informatics, Saarbrücken 66123, Germany

**Keywords:** Cognitive neuroscience, Attention

## Abstract

We provide an open access multimodal brain-imaging dataset of simultaneous electroencephalography (EEG) and near-infrared spectroscopy (NIRS) recordings. Twenty-six healthy participants performed three cognitive tasks: 1) n-back (0-, 2- and 3-back), 2) discrimination/selection response task (DSR) and 3) word generation (WG) tasks. The data provided includes: 1) measured data, 2) demographic data, and 3) basic analysis results. For n-back (dataset A) and DSR tasks (dataset B), event-related potential (ERP) analysis was performed, and spatiotemporal characteristics and classification results for ‘target’ versus ‘non-target’ (dataset A) and symbol ‘O’ versus symbol ‘X’ (dataset B) are provided. Time-frequency analysis was performed to show the EEG spectral power to differentiate the task-relevant activations. Spatiotemporal characteristics of hemodynamic responses are also shown. For the WG task (dataset C), the EEG spectral power and spatiotemporal characteristics of hemodynamic responses are analyzed, and the potential merit of hybrid EEG-NIRS BCIs was validated with respect to classification accuracy. We expect that the dataset provided will facilitate performance evaluation and comparison of many neuroimaging analysis techniques.

## Background & Summary

There have been considerable advances in brain-imaging technologies over recent years^1–3^. Brain-imaging technologies fall into two broad categories: 1) structural imaging, such as computed tomography (CT) and 2) functional imaging, such as electroencephalography (EEG). Apart from invasive methods (e.g., electrocorticography), non-invasive functional imaging methods can be mainly categorized into 1) electrophysiological methods and 2) metabolic methods^4^. EEG is the most popular electrophysiological method widely used in many research areas such as neuroscience, neural rehabilitation, and brain-computer interface (BCI)^5–14^. EEG measures the neural activities with an excellent temporal resolution (within a millisecond range), but suffers from poor spatial resolution due to volume conduction, thereby resulting in resolution limits in source localization^15^. On the other hand, near-infrared spectroscopy (NIRS) is a promising tool for monitoring metabolic changes in the brain with good spatial but low temporal resolution due to the inherent hemodynamic delay^16,17^. NIRS is more robust when confronted with electrical noise and motion-based muscle activity artifacts than EEG^18^; therefore, it can be a viable alternative to EEG in a noisy environment.

Recently, simultaneous EEG and NIRS systems, so-called hybrid systems, have been gaining more attention^19–23^. NIRS is suitable for simultaneous and artifact-free recording with EEG because its near-infrared light does not interfere with the electrical EEG measurement^4^. Over the last decade, an increasing number of research approaches have focused on the use of simultaneously acquired EEG and NIRS. For this purpose, new hybrid instrumentations have also been developed from hybrid tabletop devices into miniaturized wearable instruments^24,25^. EEG-NIRS hybrids have proven their merit in a variety of research fields^19–23^. For example, hybrid EEG-NIRS BCI systems showed better classification accuracy than each unimodal BCI system^26,27^, and EEG-NIRS hybrids were also used to better understand the linguistic functions of newborns and infants^28,29^. EEG-NIRS correlation analyses helped further to reveal the intricate relationship between electrophysiological and hemodynamic changes in neuroscience^30^.

Hybrid EEG-NIRS brain-imaging techniques are extensively investigated; however, so far, the corresponding datasets are rarely released for use by other research groups—in contrast to EEG datasets^31–35^. Generally, open access datasets can save a considerable amount of time for researchers, especially when repetitive data recordings and many participants are required^36^. Furthermore, using an identical dataset allows convenient comparison of data processing methods and validation/benchmarking of newly proposed methods. Moreover, the quality and characteristics of open datasets can be validated by many researchers and thus more credible and reproducible results can be obtained.

Our group has released an open access dataset for a hybrid EEG-NIRS BCI^37^, which includes datasets for left- and right-hand motor imagery, mental arithmetic, and several types of motion artifacts. In addition, basic data processing methods for the EEG-NIRS open access dataset were given as a reference not only for hybrid BCI beginners but also experts. However, even though the open access dataset provided was acquired from many participants (twenty-nine)^37^, we could not fully reflect a variety of experimental paradigms due to time constraints of the experiment. Note that, while in general, about 2 h is almost the maximum experimental duration for each participant to keep their concentration on the experiment, our experimental time was chosen to last about 3.5 h for each participant, including the EEG-NIRS sensor attachment.

Therefore, in the current paper, we provide a second attractive open access dataset acquired from 26 new healthy participants using three different paradigms with emphasis on *cognitive tasks* for usage of interested researchers from neuroscience, neural engineering, signal processing, BCI, and machine learning. The following cognitive tasks (paradigms) are studied: n-back (dataset A), discrimination/selection response (DSR) (dataset B), and word generation (WG) (dataset C) tasks. Therefore, the dataset provided is suitable for the purpose of hybrid BCI research among others on paradigms such as event-related hybrid desynchronization (ERD)-NIRS BCI, which has been widely investigated^38^, and hybrid event-related potential (ERP)-NIRS BCI^39^. State-of-the-art signal processing methods (e.g., noise cancelling and feature extraction) and novel analysis methods for hybrid BCI can be applied to the dataset for performance validation. For cognitive scientists, by means of simultaneous data recording and analysis, our dataset is advantageous to simultaneously monitor the electrophysiological and metabolic changes of the brain, which allow for an improved robustness and reliability of the results to be obtained^40^. In this study, basically, EEG and NIRS data are analyzed separately for dataset A and B and simultaneously with respect to classification for dataset C to verify the benefit of EEG-NIRS bimodal imaging in the BCI context. In addition, further basic findings are included to increase the understanding of bimodal data processing.

## Methods

### Participants

Twenty-six right-handed healthy participants participated in this study (9 males and 17 females, average age 26.1±3.5 years (mean±standard deviation) ranging from 17 to 33 years). Please refer to the Supplementary Information for more details (see Supplementary Table S1). None of the participants reported neurological, psychiatric, or other brain-related diseases that might affect the result. All participants were informed about the experimental procedure and gave written consent prior to the experiment. They were financially reimbursed after the experiment. This study was conducted according to the Helsinki declaration and was approved by the Ethics Committee of the Institute of Psychology and Ergonomics, Berlin Institute of Technology (approval number: SH_01_20150330).

### Experimental paradigm

The participants sat on a comfortable armchair in front of a 24’ LCD monitor. The distance between the participants’ eyes and the monitor was approximately 1.2 m. The participants put their index and middle fingers on numeric keypad buttons (number 7 and 8) fixed to the right armrest. They were instructed to keep their eyes on the monitor and refrain as much as possible from moving their body throughout the data recording. The experiment consisted of three sessions of n-back (dataset A), DSR (dataset B), and WG (dataset C) task each. Because of the strain on the participants’ attention over the course of a long recording time (approx. 3.5 h), data recording was performed in descending order according to the task difficulty (dataset A→C→B).

### Dataset A: n-back

**Figure 1:**
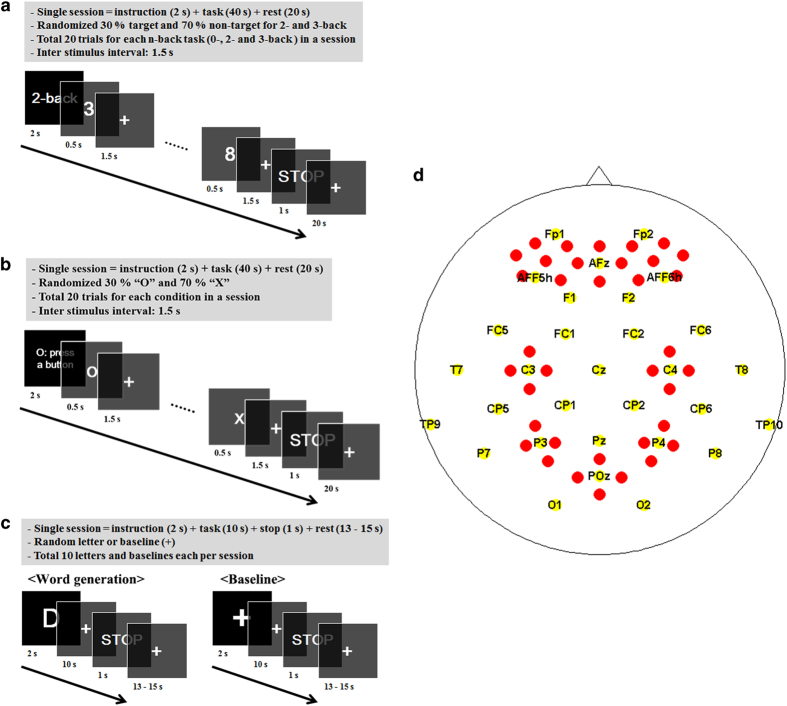
Timing sequence of the experiments: a) n-back, b) DSR task, c) WG and BL task. EEG-NIRS placement: d) EEG electrodes (yellow) and NIRS channels (red).

The dataset consisted of three sessions, where one session contained three series of 0-, 2-, and 3-back tasks in a counterbalanced order (i.e., 0→2→3→2→3→0→3→0→2). Nine series of n-back tasks were performed for each participant. A single series was composed of a 2 s instruction showing the type of the task (0-, 2- or 3-back), a 40 s task period, and a 20 s rest period. A short beep (250 ms) was provided to the participant to indicate the beginning and the end of the task period, where the word ‘STOP’ was additionally displayed on the monitor for 1 s at the end of the task period. During the rest period, a fixation cross was displayed on the monitor. Figure 1a shows the timing sequence of the n-back task. In the task period, a random one-digit number was given every 2 s. Each number was displayed for 0.5 s, followed by a fixation cross for the remaining 1.5 s. Twenty trials were repeated where the targets appeared with a 30% chance (70% non-targets). In the 0-back task, participants either pressed a ‘target’ button (number 7) with their right index finger or ‘non-target’ button (number 8) with their right middle finger. This ensured that the participants were engaging in the experiment. In the 2- or 3-back task, the participants responded by pressing the ‘target’ button if a currently displayed number matched the 2 or 3 preceding numbers, respectively, or the ‘non-target’ button. In the rest period, the fixation cross was displayed for 20 s, and the participants were asked to relax and gaze at it in order to allow the brain state to return to a baseline and prevent excessive eye movements. A total of 180 trials were performed (=20 trials×3 series×3 sessions) for each n-back task.

### Dataset B: DSR

The dataset included three sessions, where one session contained three series of DSR in a counterbalanced order. Nine series of DSR tasks were performed for each participant. A single trial involved a 2 s instruction showing ‘O: press a button’ on the monitor, a 40 s task period, and a 20 s rest period with a fixation cross in the middle of the monitor. The task period started with a short beep (250 ms) and ended with another short beep (250 ms), and ‘STOP’ displayed on the screen for 1 s. Figure 1b shows the timing sequence of the DSR task. In the task period, the symbol ‘O’ and the symbol ‘X’ were given every 2 s in a random order. Every symbol was displayed for 0.5 s and the fixation cross was shown for the remaining 1.5 s. Twenty trials were repeated where the symbol ‘O’ appeared at a 30% chance (70% symbol ‘X’). The participants responded by pressing the ‘target’ button (number 7) with their right index finger as soon as the symbol ‘O’ was displayed. The participants pressed ‘non-target’ button (number 8) with their right middle finger if the symbol ‘X’ was displayed to ensure them to focus on the experiment consistently, and the response time did not matter. In the rest period, the participant did the same as the n-back task, relaxing and gazing on the fixation cross. As with the n-back task, a total of 180 trials were performed (=20 trials×3 series×3 sessions).

### Dataset C: WG

The dataset has three sessions for each participant, where each session contains ten trials of WG and baseline (BL) tasks each. Note that unlike datasets A and B, dataset C is not composed of series of stimuli but temporally separated trials. In total, twenty trials (ten WG and ten BL) were repeated at random in each session. A single trial contained a 2 s instruction showing an initial single letter for WG or the fixation cross for BL, a 10 s task period, and a 13–15 s rest period with the fixation cross in the middle of the monitor. The task period started with a short beep (250 ms) and fixation cross displayed in the middle of the monitor, and ended with another short beep (250 ms) and a ‘STOP’ sign displayed on the monitor for 1 s. Figure 1c shows the timing sequence of WG. For WG in the task period, the participants were instructed to keep thinking of words beginning with the letter that was previously given as fast as possible. Repetition of the same word was not allowed in order to prevent the participants from becoming accustomed to the problems. For BL, they were asked to relax and gaze at the fixation cross for a low cognitive load. In the rest period, the participants did the same as in the rest period of the n-back task. A total of 60 trials were performed (30 trials (=10 trials×3 sessions) for WG and 30 trials for BL).

### Data acquisition

All EEG and NIRS signals were recorded simultaneously. For timing synchronization, triggers were sent to both EEG and NIRS equipment simultaneously via a parallel port using MATLAB.

EEG data was recorded using a multichannel BrainAmp EEG amplifier (Brain Products GmbH, Gilching, Germany) at a sampling rate of 1,000 Hz. Thirty EEG active electrodes were placed on a stretchy fabric cap (EASYCAP GmbH, Herrsching am Ammersee, Germany) according to the international 10-5 system^41^ (Fp1, Fp2, AFF5h, AFF6h, AFz, F1, F2, FC1, FC2, FC5, FC6, Cz, C3, C4, T7, T8, CP1, CP2, CP5, CP6, Pz, P3, P4, P7, P8, POz, O1, O2, TP9 (reference) and TP10 (ground)). The EEG amplifier was also used to measure the electrooculogram (EOG). EOG was recorded using two vertical (above and below the right eye) and two horizontal (outer canthus of each eye) disposable electrodes at the same sampling rate as the EEG data.

NIRS data was acquired with a NIRScout (NIRx Medizintechnik GmbH, Berlin, Germany) at a sampling rate of 10.4 Hz. Sixteen sources and sixteen detectors were placed at frontal (sixteen channels around AFz, AF3, AF4, AF7 and AF8), motor (four channels each around C3 and C4), parietal (four channels each around P3 and P4), and occipital (four channels around POz) areas. An adjacent source-detector pair configures a NIRS channel; 36 channels were configured. The NIRS channels, each of which was composed of a pair of a source and a detector, were created around AFpz, AFp3, AFp4, AFp7, AFp8, AF1, AF2, AF5h, AF6h, AF7, AF8, AFFz, AFF3h, AFF4h, AFF5h, AFF6, FCC3, FCC4, C3h, C4h, C5h, C6h, CCP3, CCP4, CPP3, CPP4, P3h, P4h, P5h, P6h, PPOz, PPO3, PPO4, PO1, PO2, and POOz according to the international 10-5 system. The source-detector distance was set to 30 mm for all the channels^42–44^. NIRS optodes were fixed on the same cap as the EEG electrodes. Figure 1d shows the locations of the EEG and NIRS channels. Yellow and red circles denote the location of the EEG and NIRS channels, respectively. EEG electrodes are relatively uniform in their distribution over the whole head. Reference and ground electrodes are located at TP9 and TP10, respectively. NIRS channels are located on (pre)frontal (sixteen channels), motor (four channels around C3 and C4 each), parietal (four channels around P3 and P4 each), and occipital areas (four channels around POz).

### Data processing

All data processing was done using MATLAB R2013b (MathWorks, Natick, MA, USA). For the EEG data, EEGLAB was used for automatic artifact removal (AAR) toolbox to gain ocular artifact rejection, and the BBCI toolbox was used for further data processing^45–51^. Specifications of the data processing are summarized in Tables 1–4.

The raw EEG data was downsampled to 200 Hz and band-pass filtered (6th order zero-phase Butterworth filter) with a passband of 1–40 Hz. Improved weight-adjusted second-order blind identification (iWASOBI) method implemented in AAR toolbox was applied to the EEG data to eliminate ocular artifacts^52,53^. Event-related (de)synchronization (ERD/ERS) analysis was done for all datasets. Because the control conditions, such as 0-back or symbol 'X', were not included in dataset C, it is impossible to distinguish between the control and task-relevant ERPs. Therefore, ERP analysis was done for datasets A and B only. For the ERD/ERS analysis, ocular artifact-free EEG data was segmented into epochs ranging from −5 s to the end of rest period (datasets A and B: to 60 s/dataset C: to 25 s). Note that 0 s indicates the onset of the task period. Baseline correction was performed by subtracting the average value of epochs ranging from −5 to −2 s. As mentioned before, because the type of the task was introduced from −2 to 0 s (2 s instruction), the relevant ERS/ERD would be elicited since then. For the ERP analysis, the ocular artifact-free EEG data segmented into epochs ranging from −0.1 to 1 s. The baseline was corrected by subtracting the average value of epochs ranging from −0.1 to 0 s.

The raw NIRS optical intensity data converted to deoxy- (HbR) and oxy hemoglobin (HbO) concentration changes (ΔHbR and ΔHbO) using the modified Beer-Lambert law^54,55^ and downsampled to 10 Hz. Since the fundamental frequencies of the datasets A and B were very low (1/60 s^−1^=0.017 Hz) and in order to avoid rejecting the fundamental frequency component, the downsampled data was low-pass filtered instead of band-pass, (6th order zero-phase Butterworth) with 0.2 Hz cut-off frequency to remove the high frequency instrument and systemic physiological noise (e.g., cardiac and respiratory)^56^. The whole filtered data was segmented into epochs ranging from −5 s to the end of the rest period (datasets A and B: to 60 s/dataset C: to 25 s).

### Classification

All classification procedures were performed using MATLAB R2013b and the BBCI toolbox. Specification for the classification procedures is summarized in Tables 2–4. Classification procedures were conducted on EEG datasets A-C and NIRS dataset C. For datasets A and B, ‘target’ versus ‘non-target’ ERP classification was solely conducted, while for dataset C, WG versus BL EEG-NIRS meta-classification was conducted^26^. Since the sampling rate of NIRS data was not enough to distinguish a task-induced fast response, such as ERP, the fast NIRS response of datasets A and B was not considered in this study. This is the reason why EEG-NIRS meta-classification was performed only for dataset C.

For ERP analysis in the datasets A and B, throughout a whole epoch (−0.1 to 1 s), participant-specific five most discriminative time periods were found by means of a heuristic procedure estimating the time intervals with the highest absolute *r-value*^57^. The *r-value* indicates the separability between ‘target’ and ‘non-target’ conditions. Features were extracted using the mean values of 5 selected time intervals (dimension: 28 channels×5 time intervals). A shrinkage linear discriminant analysis (sLDA) was applied for 10×10-fold cross-validation^48,49^.

In dataset C, the participant-specific frequency band showing the best separability between WG and BL was selected. The details are shown in the Supplementary Material (see Supplementary Fig. S1). EEG features were estimated using a sliding window (window size: 5 s, step size: 1 s, 80% overlap) shifted from the beginning to the end of the epoch (−5 to 25 s). Common spatial pattern (CSP) filtering was then applied to decompose the EEG data to determine the most discriminative CSP components. Features were finally extracted using the log-variance of CSP components (dimension: 28) in descending order of a score based on median variance. This measure is more robust for outlier trials than a conventional approach based on eigenvalues^58^. The CSP components contain the most discriminative information over each time period of the sliding window. sLDA was used as a classifier. A 10×5-fold cross-validation was performed to evaluate the classification performance for each sliding window. Note that the spatial CSP filters were built based on only the training data, and then applied to the test data of the classifier for each sliding window. In dataset C, i) the mean value and ii) average slope of ΔHbR and ΔHbO were estimated using the sliding window identical to the EEG analysis for the feature vectors (dimension: 36 channels×2 chromophores). The classifier and cross-validation method were identical to the EEG classification method.

Since a fast response, such as ERP, cannot be detected by means of NIRS, a meta-classification method was applied to only dataset C in order to validate the potential merit of the combination of EEG and NIRS data. The outputs of the individual EEG and NIRS classifiers (LDA-projected data) were combined to create feature vectors for the meta-classifier. All possible combinations of EEG and NIRS chromophores (e.g., EEG+HbR, EEG+HbO, and EEG+HbR+HbO) were considered. The cross validation of the meta-classifier followed the same procedure as for the aforementioned EEG and NIRS classifications.

### Code availability

The MATLAB scripts used to generate figures and tables included in this paper are available at https://github.com/JaeyoungShin/simultaneous_EEG-NIRS.

## Data Records

The data in vendor-agnostic and vendor-specific formats are freely downloadable from the open access repository (Data Citation 1) and website (http://doc.ml.tu-berlin.de/simultaneous_EEG_NIRS/). The MATLAB-compatible resource (in vendor-agnostic format) consists of EEG/EOG data and NIRS data separately (a total of approx. 6.41 GB). The name of each zip file consists of participant code and modality, e.g., ‘VP001-EEG’ for EEG data and ‘VP001-NIRS’ for NIRS data. Each zip file contains individual datasets A-C, and has continuous data (cnt), marker (mrk), and montage (mnt) for datasets A-C each. Note that cnt, mrk, and mnt files have suffixes corresponding to each dataset (A: _nback, B: _dsr, and C: _wc). Each file comprises of MATLAB structure array with several fields. For NIRS data, the cnt files contain deoxy/oxy-hemoglobin data as separate fields. For data structure information, please refer to the BBCI toolbox^45,59^. The description text file of the uploaded dataset explains the data structure in more detail. The rawdata in vendor-specific format are also provided without any preprocessing or conversion (Data Citation 1).

## Technical Validation

In datasets A and B, grand average ERPs over all participants are provided based solely on correct responses. Wrong or missed responses (pressing a wrong button or no response, respectively) were excluded. Behavioral data (e.g., reaction time) can be found in Tables 5 and 6.

### Dataset A: n-back task—ERP

**Figure 2:**
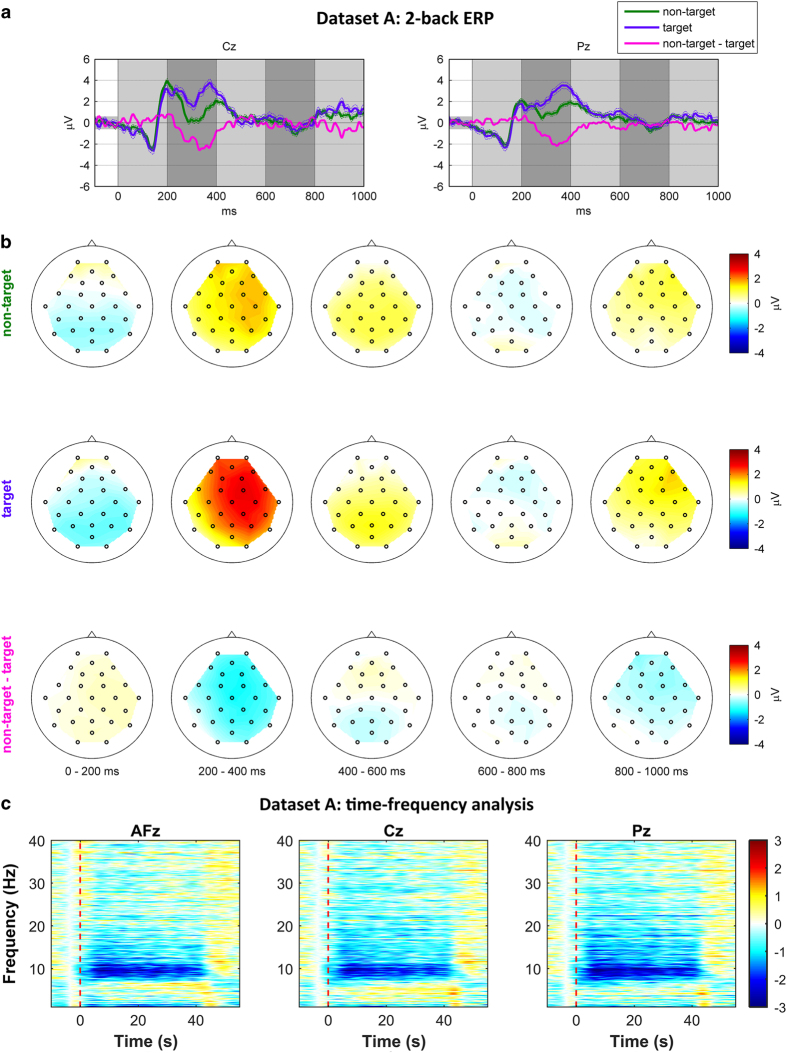
Grand average ERP waveforms (a), spatial distribution of amplitudes over scalp (b), and spectral power (c)(unit: dB) for the 2-back task. Vertical red dashed lines indicate the task onset.

Figure 2a shows the grand average waveforms of ERPs for the 2-back task at two midline locations (Cz and Pz). Upper and lower bounds (dashed lines) indicate standard errors of the grand average. A small gray patch before 0 s indicates the baseline correction period, and gray shades after 0 s depict time periods corresponding to each of the scalp plots below. In the waveforms, N100 and P200 are elicited by auditory (short beep) and visual (displayed number) stimuli, respectively, which are followed by a delayed P300 around 400 ms. Note that the largest difference between both conditions (‘non-target-target’) is observed at 0.2–0.4 s. Figure 2b shows the scalp plots for ‘non-target,’ ‘target,’ and a ‘non-target-target,’ respectively. In the scalp plots, very large positive (red) ERPs are mainly shown over frontal, central, and parietal areas at 200–400 ms for target. For the 3-back task, refer to the Supplementary Information (Supplementary Fig. S2).

Table 5 shows the summary of the ERP analysis results for the n-back task. As can be seen in Table 5, the percentage of correct responses decreases (Wilcoxon signed rank sum test, *P*<0.01) and the reaction time increases (Wilcoxon signed rank sum test, *P*<0.01) as the cognitive load increases (2-back versus 3-back). However, the classification accuracy does not significantly differ between 2-back and 3-back tasks (Wilcoxon signed rank sum test, *P* > 0.05).

### Dataset A: n-back task—ERD/ERS

Figure 2c shows the grand average of EEG spectral power in the dB scale for the 2-back task at three midline locations AFz, Cz, and Pz. For the 2-back task, distinct ERD in the alpha and week ERD in the low beta band are observed overall at all three locations over the frequency range of interest (0–40 Hz) during the task period (0–40 s). For 0-back and 3-back tasks, refer to the Supplementary Information (Supplementary Fig. S3).

### Dataset A: n-back task—NIRS

**Figure 3:**
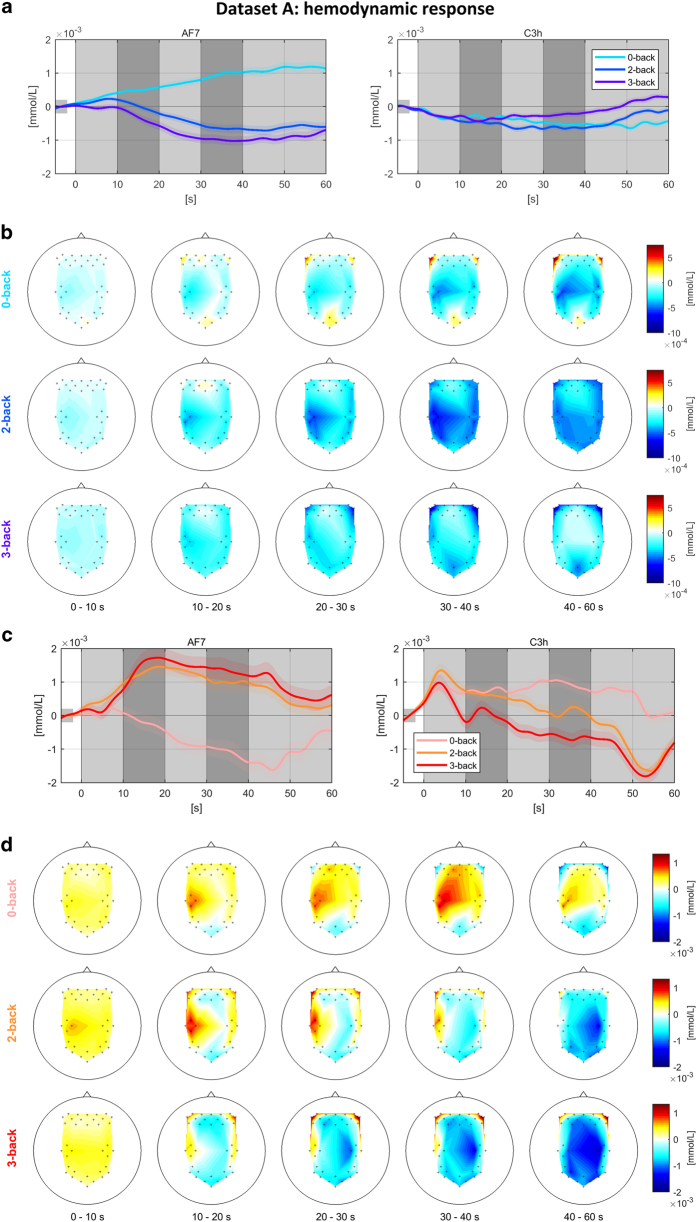
Grand average time courses of HbR (a) and HbO (b) at NIRS channels for 0-, 2-, and 3-back tasks around AF7 and C3h and spatial distribution of HbR (b) and HbO (d) amplitudes over scalp. Colorbar indicates the concentration changes of NIRS chromophores (unit: mmol/L). Green dots in scalp plots denote channel locations (frontal: AF7 and occipital: C3h).

Figure 3a and c depict the grand average time courses of HbR and HbO, respectively, for n-back tasks at two locations (around AF7 and C3h) representing prefrontal and parietal/occipital areas, and Fig. 3b and d depict scalp plots for HbR and HbO, respectively. In Fig. 3b, for the 0-back task, HbR increases in the prefrontal and occipital areas, but decreases in the frontal and motor areas. Note that the strong HbR increases are shown at the left and right lateral channels in the prefrontal area. For 2- and 3-back tasks, HbR decreases in the bilateral prefrontal, motor, parietal, and occipital areas. More distinct decreases are detected as the task difficulty increases (2-back <3-back) in the left and right lateral prefrontal areas. On the other hand, in motor and parietal areas, HbR decrease was observed more clearly while performing the 2-back task. In Fig. 3c and d, the opposite trends are seen compared with Fig. 3a and b. For the 0-back task, HbO increases in the mid-frontal, motor, and parietal areas while it decreases in the prefrontal and occipital areas. Note that a stronger HbO increase is shown in the left motor area than in the other side. For the 2- and 3-back tasks, HbO decreases in the mid-frontal, parietal, and occipital areas while it increases in the left and right lateral prefrontal and motor areas. Stronger increases are shown as the task difficulty increases (2-back <3-back) in the left and right lateral prefrontal areas. Note that the HbR and HbO do not completely go back to a baseline level after the end of the task period. It might be caused by the relatively short rest period (20 s) compared to the task period (40 s).

### Dataset B: DSR—ERP

**Figure 4:**
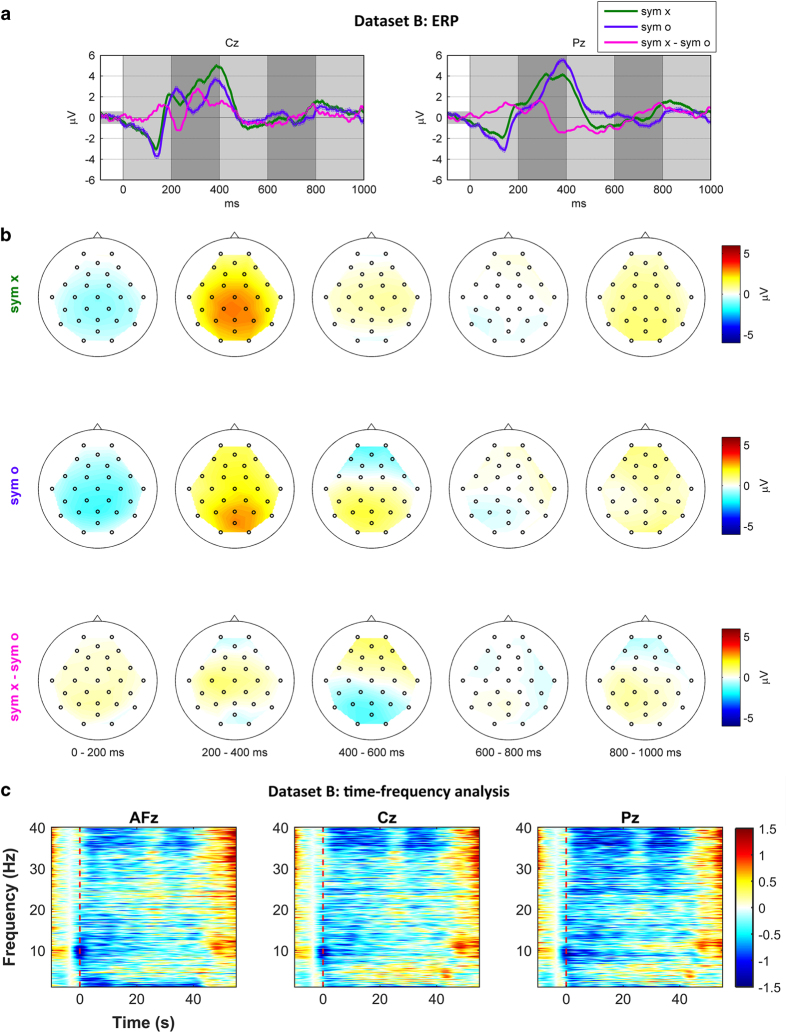
Grand average ERP waveforms (a), spatial distribution over scalp (b), and spectral power (unit: dB) for the DSR task (c). Vertical red dashed lines indicate the task onset.

Figure 4a and b show the grand average waveforms of ERPs for symbols ‘O’ and ‘X’ at two midline locations (Cz and Pz) and scalp plots for symbol ‘X’, symbol ‘O’, and a symbol ‘X-symbol ‘O’. Upper and lower bounds (dashed lines) indicate standard errors of the grand average. In the waveforms, N100 and P200 are clearly elicited by auditory (short beep) and visual (displayed number) stimuli, respectively, and the delayed P300 is observed around 400 ms. Delayed P300 amplitude for the symbol ‘X’ is larger than that for the symbol ‘O’ at Cz while it is the other way around at Pz. In the scalp plot of symbol ‘X-symbol ‘O’, the amplitude of the delayed P300 for symbol ‘X’ is larger than that for symbol ‘O’ in the frontal area while smaller in the parietal and occipital areas (400–600 ms).

Table 6 shows the summary of the ERP analysis results for the DSR task. The percentage of correct responses is almost the same (Wilcoxon signed rank sum test, *P*>0.05). A classification accuracy of 86.8±5.9% is achieved to differentiate the symbol ‘O’ from the symbol ‘X’ using ERP.

### Dataset B: DSR—ERD/ERS

Figure 4c shows the grand average of EEG spectral power in the dB scale for the DSR task at three midline locations (AFz, Cz, and Pz). During the task period (0–40 s), the gamma band ERD (>30 Hz) commonly appears over the three channels, especially strongly in the parietal area (Pz). Weak alpha band ERD is observed in the parietal area (Pz), and delta band ERD in the frontal area (AFz) is clearly seen.

### Dataset B: DSR—NIRS

**Figure 5:**
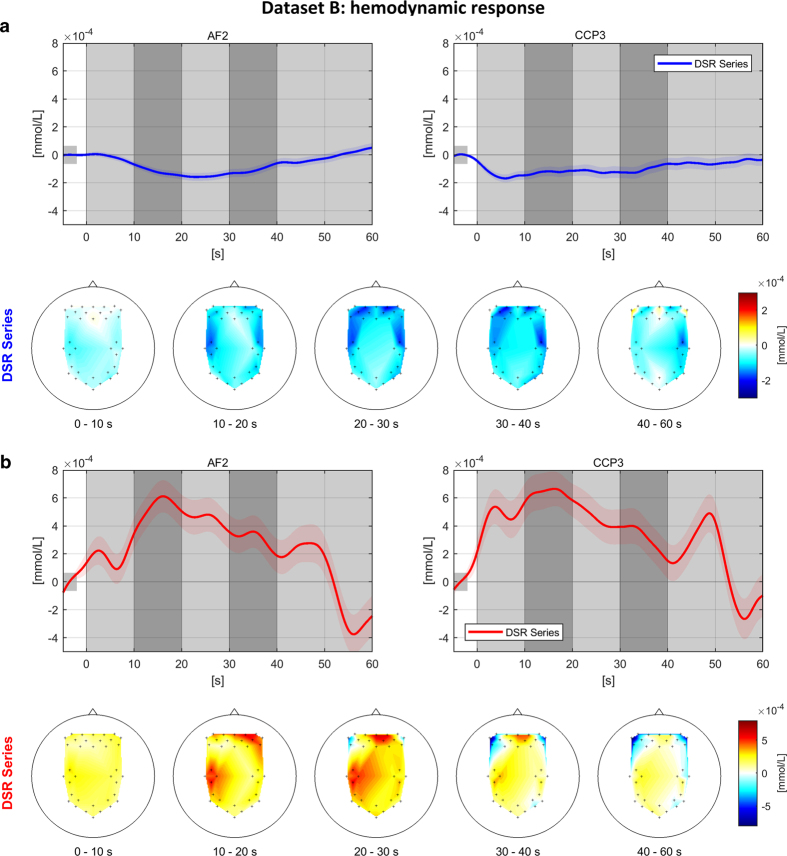
Grand average time courses of HbR (a, top) and HbO (b, top) for the DSR task around AF2 and CCP3 and spatial distribution of corresponding amplitudes over scalp (a,b, bottom). Colorbar indicates the concentration changes of NIRS chromophores (unit: mmol/L). Green dots in scalp plots denote channel locations (frontal: AF2 and motor: CCP3).

Figure 5a and b show the grand average time courses of HbR and HbO, respectively, at two locations (AF2 and CCP3) representing prefrontal and motor/parietal areas and their scalp plots, respectively. In Fig. 5a, HbR decreases in the frontal and motor areas and normally reaches the minimum at 10–20 or 20–30 s. In Fig. 5b, the opposite trends are observed. In contrast with the n-back task, the time courses nearly go back to the baseline level.

### Dataset C: WG—ERD/ERS

**Figure 6:**
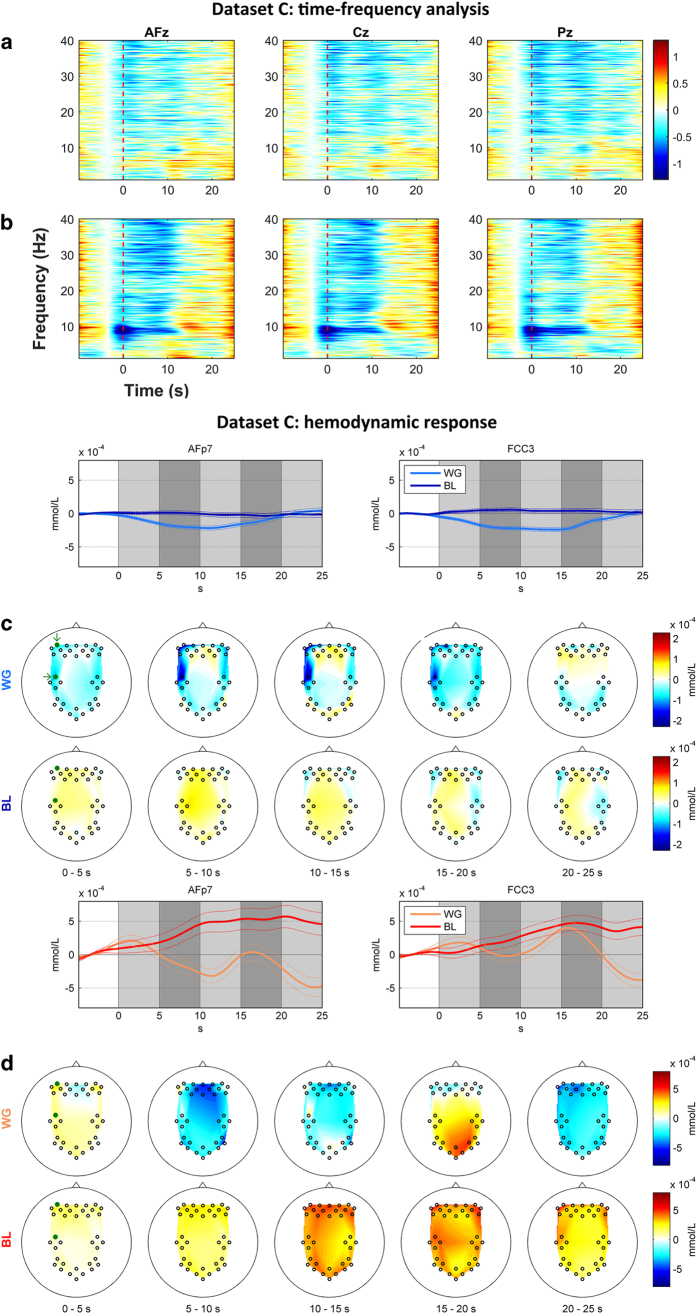
Grand average of the EEG spectral power (unit: dB) during BL (a) and WG (b); time courses of HbR (c, top) and HbO (d, top) for the WG task around AFp7 and FCC3 and corresponding spatial distribution of amplitudes (c,d, bottom). Vertical red dashed lines indicate the task onset. Green dots in scalp plots denote channel locations (frontal: AFp7 and motor: FCC3).

Figure 6a and b show the grand average of EEG spectral power in the dB scale, for BL and WG, respectively, at three midline locations (AFz, Cz, and Pz). For BL (Fig. 6a), no significant change was observed during task period over the whole areas. For WG (Fig. 6b), distinct ERD can be identified in beta and gamma band during the task period in the frontal and central areas. The alpha band ERD (around 10 Hz) is clearly observed in all three channels. The distinct ERD over three channels before the task onset (0 s) is observed in the alpha band (around 10 Hz), which is thought to be due to the recognition of the initial single letter for WG.

### Dataset C: WG—NIRS

Figure 6c and d show the grand average time courses of HbR and HbO, respectively, at two locations (around AFp7 and FCC3) representing prefrontal and motor areas and their scalp plots. In Fig. 6c, for WG, during the task period (0–10 s), the HbR markedly decreases in the bilateral prefrontal area. Significant decrease in the left motor cortex is also observed, and the reduced HbR returns to baseline about 5 s after the task period is over. Whereas, for BL, slight HbR increase is commonly seen over whole areas along the time. In addition, slight HbR decrease is observed after the task period in the bilateral prefrontal and right motor areas. Particularly, AFp7 and FCC3 show little change in the time course of the hemodynamic response. In Fig. 6d, for WG, in the prefrontal midline channels, the HbO decrease is observed up to 10 s and then returned to baseline. In the motor, parietal, and occipital areas, the HbO decreases during the task period and starts to increase after the task period up to 20 s. Strong HbO increase is particularly observed in parietal and occipital areas in 15–20 s. After 20 s, the HbO decrease throughout the whole areas is detected. For BL, the HbO increases gradually along the time by 20 s while it decreases slightly since then.

### Dataset C: WG—hybrid

**Figure 7:**
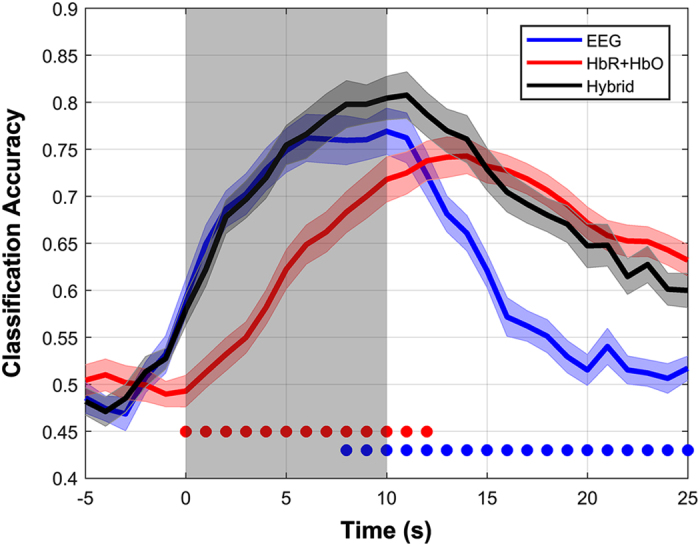
EEG, NIRS (HbR+HbO), and hybrid classification accuracies over time estimated by the sliding window.

Figure 7 shows the EEG, NIRS, and meta-classification accuracies over time estimated by the sliding window. The x-axis presents the right end of the sliding window. The y-axis indicates the classification accuracy. EEG classification accuracy starts to rise in advance of the onset of the task period. It is presumed that the initial task condition was given at t=−2 s, then the participants recognized the beginning of the task and began to think what to do in the task period. EEG classification accuracy peaks at t=10 s, scoring 76.9% classification accuracy and decreases after that. NIRS (HbR+HbO) classification accuracy starts to increase from the task onset and peaks at t=14 s scoring 74.3% classification accuracy and decreases afterwards, but it does not completely return to its base level. It may happen that the HbR and HbO do not return to the baseline completely due to the insufficient length of the rest period. Nevertheless, it does not influence the NIRS classification accuracy by degrading the classification performance seriously. The hybrid classification accuracy achieved the highest classification accuracy (80.7%) at t=11 s. Circles below the figure indicate the time periods at which the hybrid approach outperforms EEG (blue) and NIRS (red) significantly (Friedman test: *P*<0.05, *post hoc*: Wilcoxon signed rank sum test, false discovery rate-adjusted *P*<0.05)^60^. The hybrid system is capable of enhancing the classification accuracy better than both EEG and NIRS and reducing the delay to be shorter than NIRS. After t=13 s, no significant improvement in classification accuracy is achieved by means of the hybrid system since EEG shows very poor classification and may not contribute in improving the hybrid classification accuracy.

## Usage Notes

A limit of all datasets available is the time constraint within every experiment and the desire to study a variety of paradigms. Thus, typically the rest periods for the NIRS data are not long enough to let the response return to the baseline level. We used an ISI of 1.5 s for n-back (dataset A) and DSR (dataset B) tasks. Considering that the peak hemodynamic responses can be observed about 6 s after a stimulus is presented^61^, the n-back and DSR dataset measured with the short ISI is not appropriate for event-related NIRS analysis. However, as can be particularly seen in Figs. 6c, d and 7 showing the event-related hemodynamic response and classification accuracy over the time for WG task (dataset C), it is revealed that the relatively short rest period (ISI) only insignificantly affects both the hemodynamic responses and overall performance. Therefore, instead of thinking of this particular aspect as a drawback in the presented data, we consider it as a challenge to data analysis techniques that all experiments involving NIRS will inevitably have to face. In that sense, our data set provides an excellent testbed for exploring means to tackle this challenge.

A further limit of our recordings is that it was conducted within a day, and thereby the long term test-retest reliability cannot be validated. In addition, because the experiment was carried out for a long time (approx. 3.5 h), the participants’ fatigue may potentially have an influence on the performance. Also, during WG, because we did not assess in detail how well and accurately the participant performed the given task inter-participant variability of the task performance cannot be well studied using this dataset. Potential users, therefore, should consider this issue when employing and analyzing our dataset. Thus, further novel open data sets addressing this important aspect would be welcome to the community.

Despite the several limitations, we believe that our open access hybrid dataset of EEG and NIRS may be useful to address neuroscientific challenges of the various paradigms, e.g., ERD/S, ERP, visual/auditory evoked potential (V/AEP), and rapid serial visual presentation (RSVP), thereby advancing our understanding of neural information processing as well as neurovascular coupling. Our hybrid dataset can be also exploited to advance BCI technology with respect to classification performance. In this study, the fusion of EEG and NIRS data was performed on the classification stage, but it could alternatively be done in an early stage (i.e., feature extraction) by e.g. extracting highly correlated components between electrophysiological and metabolic signals^21^. Research in this direction may thus further improve the classification performance of hybrid BCI systems.

Some of useful MATLAB functions and demo scripts for analyzing EEG and NIRS data are available in the BBCI toolbox (https://github.com/bbci/bbci_public). This toolbox is optimized to support file formats for Brain Products (http://www.brainproducts.com/) and NIRx (http://nirx.net) equipment.

## Additional information

**How to cite this article:** Shin, J. *et al.* Simultaneous acquisition of EEG and NIRS during cognitive tasks for an open access dataset. *Sci. Data* 5:180003 doi: 10.1038/sdata.2018.3 (2018).

**Publisher’s note:** Springer Nature remains neutral with regard to jurisdictional claims in published maps and institutional affiliations.

## Supplementary Material



Supplementary Information

## Figures and Tables

**Table 1:** Specification for EEG data processing.

***EEG***	**Dataset A**	**Dataset B**	**Dataset C**
task	n-back	discrimination/selection response	word generation
downsampling	200 Hz	200 Hz	200 Hz
filtering	1–40 Hz	1–40 Hz	1–40 Hz
ocular artifact elimination*	○	○	○
ERD/ERS analysis	○	○	○
ERP analysis	○	○	×

*ocular artifact elimination by AAR toolbox.

**Table 2:** Specification for ERD/ERS analysis.

***ERD/ERS analysis***	**Dataset A**	**Dataset B**	**Dataset C**
epoch	−5 to 60 s	−5 to 60 s	−5 to 25 s
baseline correction	−5 to −2 s	−5 to −2 s	−5 to −2 s
classifier	×	×	sLDA
cross validation	×	×	10×5-fold
CSP range*	×	×	−2 to 10 s

*epoch range used to estimate participant-dependent frequency band for CSP.

**Table 3:** Specification for ERP analysis.

***ERP analysis***	**Dataset A**	**Dataset B**	**Dataset C**
epoch	−0.1 to 1 s	−0.1 to 1 s	×
baseline correction	−0.1 to 0 s	−0.1 to 0 s	×
classification	sLDA	sLDA	×
cross validation	10×10-fold	10×10-fold	×

**Table 4:** Specification for NIRS data processing.

***NIRS***	**Dataset A**	**Dataset B**	**Dataset C**
task	n-back	discrimination/selection response	word generation
downsampling	10 Hz	10 Hz	10 Hz
filtering	low pass 0.2 Hz	low pass 0.2 Hz	low pass 0.2 Hz
ocular artifact elimination	×	×	×
epoch	−5 to 60 s	−5 to 60 s	−5 to 25 s
baseline correction	−5 to −2 s	−5 to −2 s	−5 to −2 s
classifier	×	×	sLDA
cross validation	×	×	10×5-fold

**Table 5:** Summary of ERP analysis results for the n-back task.

**Results**	**0-back**	**2-back**	**3-back**
Correct responses (%)	99.0±1.8	88.1±11.1	73.5±14.5
Wrong responses (%)	—	11.5±11.0	25.7±14.7
Missed responses (%)	1.0±1.8	0.4±0.8	0.8±1.4
Reaction time (ms)	359±114	761±201	833±195
Classification acc. ±std (%) (versus 0-back)	—	76.9±8.0	76.0±7.9
Note the values are shown in mean±standard deviation.			

**Table 6:** Summary of ERP analysis results for the DSR task.

**Results**	**symbol ‘X’**	**symbol ‘O’**
Correct responses (%)	99.6±0.8	99.4±1.0
Reaction time (ms)	—	415±53
Classification acc. ±std (%) (versus symbol ‘X’)	—	86.8±5.9
Note the values are shown in mean±standard deviation.		
