# Supplementary material for: Simultaneous acquisition of EEG and NIRS during cognitive tasks for an open access dataset
Source: Sci Data. 2018 Feb 13;5:180003. doi: 10.1038/sdata.2018.3 (PMC5810421; doi:10.1038/sdata.2018.3)
Supplement: Supplementary Information [file sdata20183-s2.docx]

Simultaneous acquisition of EEG and NIRS during cognitive tasks for an open access dataset

Jaeyoung Shin^1^, Alexander von Lühmann^2^, Do-Won Kim^3^, Jan Mehnert^4^, Han-Jeong Hwang^5*^ and Klaus-Robert Müller^2,6,7*^

^1^Department of Biomedical Engineering, Hanyang University, Seoul, Korea

^2^Machine Learning Group, Berlin Institute of Technology, Berlin, Germany

^3^Department of Biomedical Engineering, Chonnam National University, Yeosu, Korea

^4^Institute of Systems Neuroscience, Medical Center Hamburg-Eppendorf, Hamburg, Germany

^5^Department of Medical IT Convergence Engineering, Kumoh National Institute of Technology, Gumi, Korea

^6^Department of Brain and Cognitive Engineering, Korea University, Seoul, Korea

^7^Max Planck Institute for Informatics, Saarbrücken, Germany

*Corresponding Author

# Supplementary information

## Demographic data

Here, we provide the demographic information (Table S1). Twenty-six participants took part in the experiment. We collected their age, gender, and dominant hand.

## Selected frequency band for WG vs BL classification by the heuristic procedure

The participant-specific frequency bands showing the best separability between WG and BL are shown in Figure S1. These frequency bands were estimated by the heuristic procedure^1^.

## Dataset A: n-back task – 3-back ERP

Figure S2a shows the grand average waveforms of ERPs for the 3-back task at two midline locations (Cz and Pz). Upper and lower bounds (dotted lines) indicate standard errors of the grand average. A small gray patch before 0 s indicates the baseline correction period, and gray shades after 0 s depict time periods corresponding to each scalp plot below. Figure S2b shows the scalp plots for “non-target,” “target,” and a “non-target - target,” respectively. A similar characteristic is observed compared to that of the 2-back task except large negative (blue) and positive (red) ERPs on the prefrontal area at 600-800 ms and 800-1000 ms, respectively, for both target and non-target.

## Dataset A: 0-back and 3-back tasks - ERD/ERS

For the 0-back task (Figure S3a), distinct EEG power variation is not observed over the frequency range of interest (0-40 Hz) during the task period (0-40 s) while distinct ERD in the alpha and week ERD in the low beta band are observed overall at all three locations for the 3-back task (Figure S3b).

**Table S1**. Demographic data including age, gender, and dominant hand (mean ± standard deviation)

| Participant | Age | Gender | Handedness |
| --- | --- | --- | --- |
| 1 | 26 | female | Right-handed |
| 2 | 26 | female |  |
| 3 | 25 | female |  |
| 4 | 30 | male |  |
| 5 | 26 | female |  |
| 6 | 25 | female |  |
| 7 | 32 | female |  |
| 8 | 24 | female |  |
| 9 | 27 | female |  |
| 10 | 22 | male |  |
| 11 | 27 | male |  |
| 12 | 33 | male |  |
| 13 | 27 | male |  |
| 14 | 22 | female |  |
| 15 | 23 | male |  |
| 16 | 30 | male |  |
| 17 | 17 | female |  |
| 18 | 25 | female |  |
| 19 | 25 | female |  |
| 20 | 26 | male |  |
| 21 | 28 | male |  |
| 22 | 22 | female |  |
| 23 | 28 | female |  |
| 24 | 31 | female |  |
| 25 | 29 | female |  |
| 26 | 22 | female |  |
| Average | 26.1 ± 3.5 | M: 9, F: 17 | Right: 26, Left: 0 |


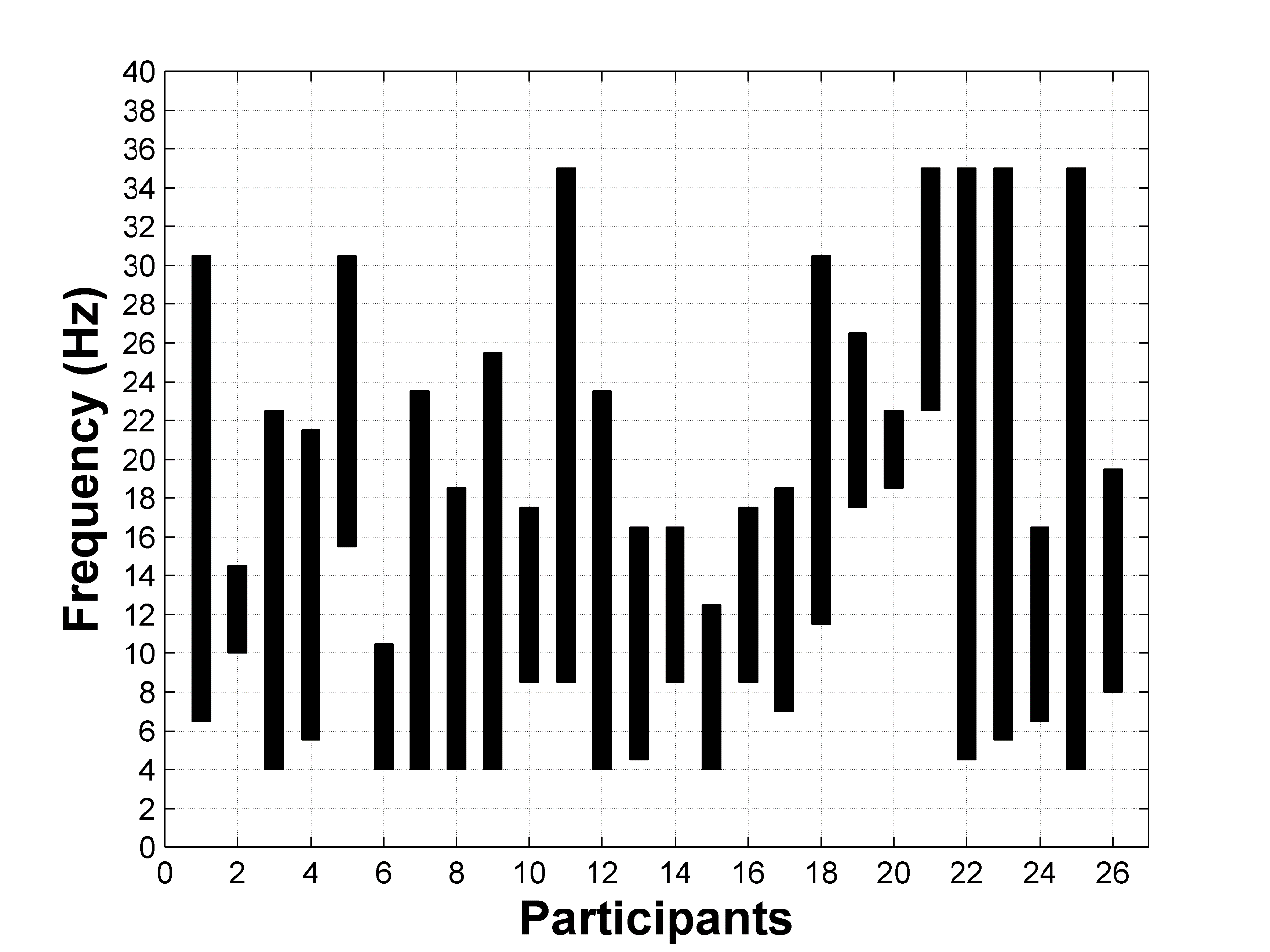


**Figure S1.** Participant-specific frequency bands showing the best separability between WG and BL estimated by the heuristic procedure.


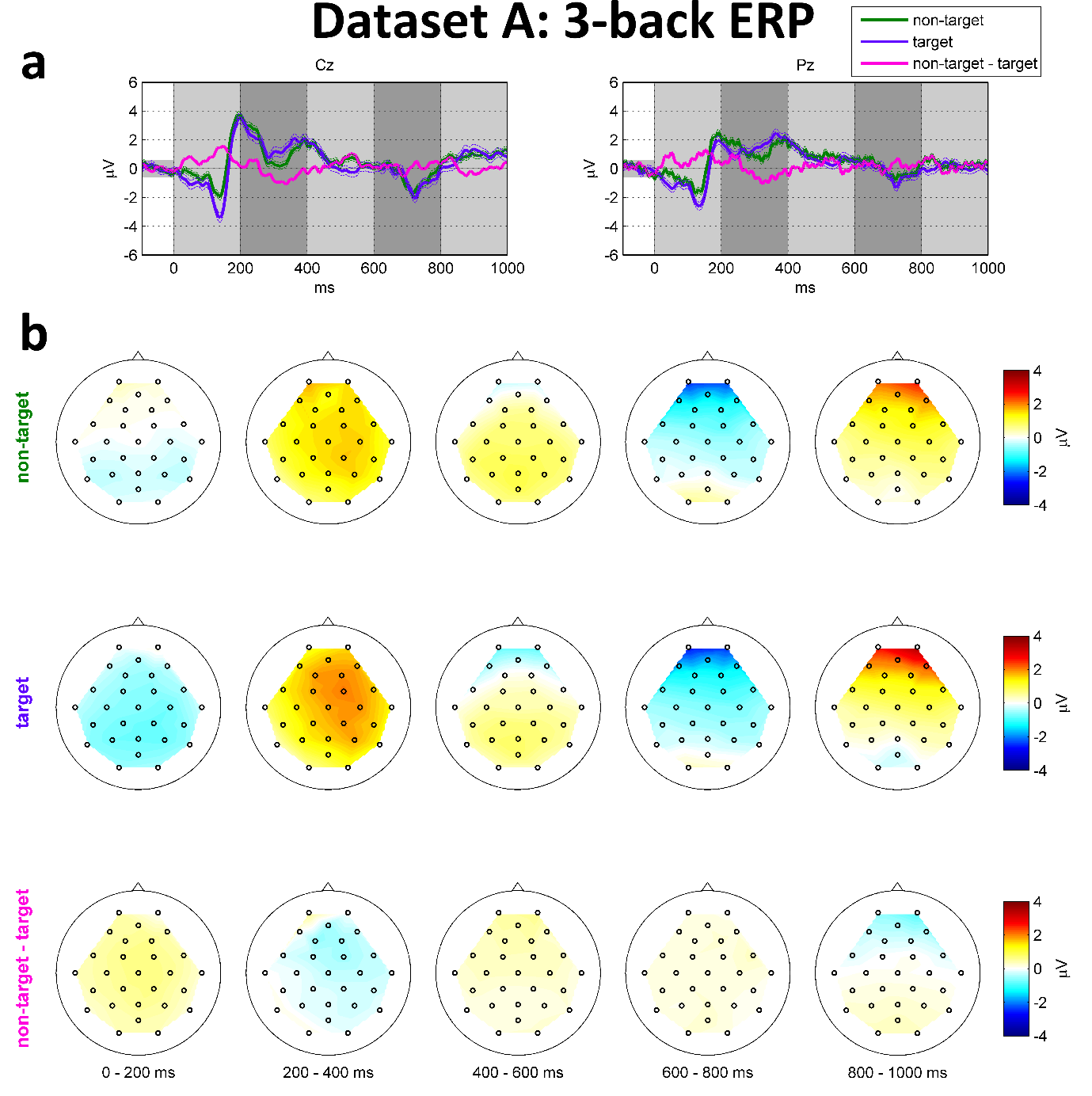


**Figure S2.** Grand average ERP waveforms and spatial distribution of amplitude over scalp for the 3-back task.


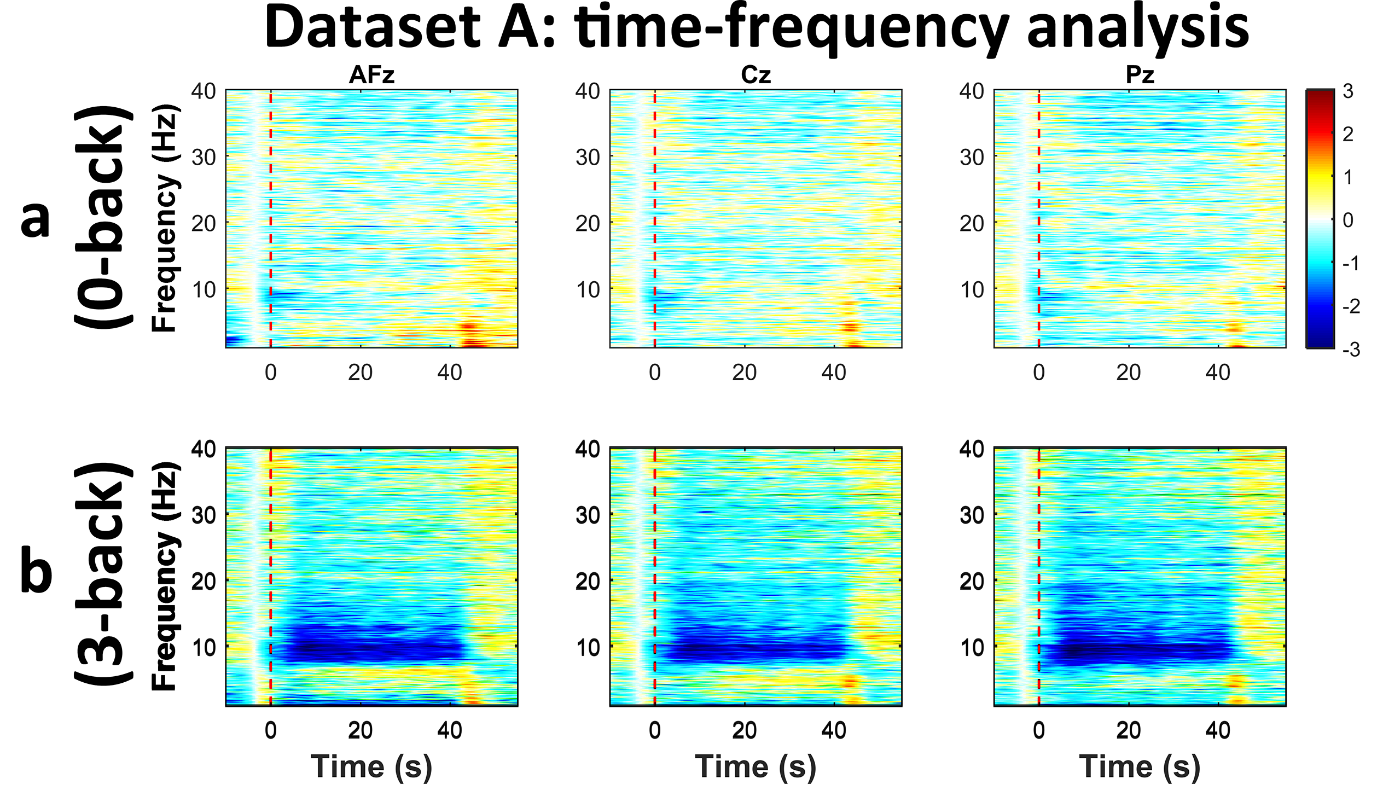


**Figure S3.** Grand average of the EEG spectral power (unit: dB) for 0-back and 3-back tasks. Vertical red dashed lines indicate the task onset.

# Reference

1. Blankertz, B., Tomioka, R., Lemm, S., Kawanabe, M. & Müller, K.-R. Optimizing spatial filters for robust EEG single-trial analysis. *IEEE Signal Process. Mag.* **25**, 41-56, (2008).
